# Light and myopia: a focus on the expanding role of non-visual opsins

**DOI:** 10.1186/s40662-025-00472-y

**Published:** 2026-01-31

**Authors:** Kate Gettinger, Kazuo Tsubota, Kazuno Negishi, Toshihide Kurihara

**Affiliations:** 1 Laboratory of Photobiology, Keio University School of Medicine, Tokyo, 160-8582 Japan; 2 Department of Ophthalmology, Keio University School of Medicine, Tokyo, 160-8582 Japan; 3 Tsubota Laboratory, Inc., Keio Hospital, 35 Shinanomachi, Shinjuku-ku, Tokyo, 160-8582 Japan

**Keywords:** Non-visual opsins, Opsins, Myopia, Myopia prevention, Unconventional opsins

## Abstract

Myopia, or near-sightedness, is a growing global concern as its incidence rate continues to dramatically rise. It has been linked to significant ocular morbidity and reduced quality of life. Despite this, much is still largely unknown about the development of and the mechanisms driving the pathogenesis of myopia. As such, myopia prevention and myopia mitigation treatment strategies are occasionally ineffective, can be difficult to adhere to, and have diminishing returns over time. Recently, non-visual opsins (OPN3, OPN4, and OPN5) have emerged as potentially impacting myopia regulation. This narrative review aims to summarize the current understanding of the non-visual opsins and how they might influence myopia. In addition, this review explores how utilizing this knowledge can help develop promising future treatment strategies to reduce the incidence and severity of myopia.

## Background

Myopia, also known as near-sightedness, has been recently projected to affect nearly 40% of the world’s population [1]. The global prevalence of adolescent myopia, specifically, is also expected to affect about a third of the population [1]. It has been estimated for the year 2015 that the annual global potential productivity loss due to visual impairment resulting from uncorrected myopia may be as high as $244 billion dollars, with East Asia bearing the greatest burden [2]. In fact, studies have indicated that in East Asian countries the prevalence of myopia among children and young adults has risen up to 90% in recent decades [3–6]. While the visual blur from myopia can be addressed with simple optical correction, high myopia can lead to visual impairment and blindness, and thus is a major health concern. Of all refractive errors, high myopia has been identified as carrying the most severe consequences [7], with visual impairment directly correlated with increasing axial length and spherical equivalent [8]. Statistically speaking, one in three individuals with high myopia will develop a severe sight-threatening complication such as myopic macular degeneration [7], optic neuropathy [9], or glaucoma [10]. Those with high myopia have also been shown to report lower quality of life and increased depression compared to those with less refractive error [11, 12].

In the wake of this, a considerable amount of effort has gone into trying to determine the mechanisms behind myopia and develop interventions to prevent and mitigate the burden of myopia. Extensive genetic analyses have attempted to identify key genes involved in myopia [13], and some genes involving light-dependent mechanisms, such as *GRIA4* [13], *Dusp4* [14], and *DRD1* [15], have emerged as likely contributors [15]. However, only a small proportion of myopia, around 18.4%, can be explained by genetic variation [16]. For example, the high myopia prevalence in East Asian populations dwindles when considering rural areas, and when analyzing the same racial populations in varying environments there are often differences in myopia prevalence [17, 18]. In addition to genetic factors, lifestyle and environment clearly contribute to myopia development. This has led to the hypothesis of a vision-dependent system of ocular growth homeostasis, where visual cues during eye development help drive emmetropization [19]. Nevertheless, there is still a notable amount of debate in regards to what factors pose the highest risk of myopia development, as well as even more debate as to what the underlying mechanisms driving myopia development may be. Some of the most agreed upon lifestyle factors influencing myopia include lack of time spent outdoors [20, 21], excessive near work [22], and high education level [23].

When focusing on the particular risk factor of time spent outdoors, outdoor lighting has emerged as one of the potential mechanisms responsible for guiding emmetropization. This has led to reconsideration of the opsin family of G-protein coupled receptors (GPCRs), especially what are referred to as non-visual opsins, or noncanonical opsins. Over time, mammals have evolved a complex set of GPCRs to detect light, and these specific receptor proteins have come to be called opsins. Opsins are light sensitive and play a role in both visual and non-visual functions. While the opsins related to visual function, namely the rod (OPN2) and cone (OPN1SW, OPN1MW, OPN1LW) opsins, have been extensively studied and appear to have a relatively straightforward role in visual perception, much less is known about the opsins related to non-visual functions. Of these, melanopsin (OPN4) has perhaps received the most research attention over recent years. Encephalopsin (OPN3) and neuropsin (OPN5), however, are also garnering more interest, with a slow and steady increase in the number of studies over the past decade [24]. Recently, their role in myopia progression and development has emerged, and it appears light and image cues may be potential contributors in the overall process of emmetropization. It is worth noting that the OPN1LW and OPN1MW opsins have also been associated with high myopia [25, 26], and OPN2 is required for normal refractive development [27], but for the purposes of this review, attention will be focused on OPN3, OPN4, and OPN5.

Of these opsins, OPN5 and rhodopsin show the highest conservation across all mammalian species, while OPN4 shows the greatest sequence diversity [28]. Regardless, all of the opsins maintain the critical amino acid residues required to sense light, giving them their ability to execute light-dependent visual and non-visual functions [28]. Due to the relative sequence conservation across mammalian species, it is suggested that studies of opsins in typical mammalian experimental models, such as mice, rats, tree shews, and primates, can directly translate to humans [24]. This makes it easier to generalize research findings and make direct correlations to clinical applications, such as in the development of new treatment strategies.

While the roles of the non-visual opsins are undergoing investigation, this narrative review aims to specifically highlight what is currently known with regards to the non-visual opsins OPN3, OPN4, and OPN5 in relation to myopia. Furthermore, we focus on the role of light-induced cues and how these opsins may not only be responsible for ocular growth and development but also might reveal promising therapeutic strategies to alleviate the global rise of myopia.

## Main text

### Myopia and light

In order to understand the relationship between non-visual opsins and myopia development, it is beneficial to review what is currently known about light input and myopia development. As mentioned previously, epidemiological studies of myopia have indicated that time spent outdoors in natural sunlight is protective against myopia development, while time spent indoors, independent of near work, is a risk factor [29–31]. High levels of ambient light have also proven protective against myopia in chick, mouse, and primate models [32–34]. A meta-review of randomized controlled trials published in 2020 considering outdoor activity as an intervention strategy for myopia found that time spent outdoors is correlated to reduced risk of myopia and slowed axial growth [35]. The reasons why outdoor activities are associated with myopia prevention are still not fully elucidated, but some proposed mechanisms include higher light intensities [36, 37], variations in the chromatic spectrum of natural light versus artificial light [38–40], differences in dioptric patterns [41], and less near focus and accommodation [42]. Due to varying study designs, it can be difficult to determine the exact correlation between the amount of time spent outdoors and the amount of myopia reduction [43].

When considering the role of the chromatic spectrum of light in myopia development, the potential role of non-visual opsins began to emerge. As it became apparent specific wavelengths of light could positively or negatively influence factors such as axial length, the spectral sensitivities of non-visual opsins began to receive more attention. But first, studies needed to establish which particular wavelengths influenced myopia development.

Early studies in chicks pointed towards blue wavelengths as being protective against myopia, while longer wavelength red light resulted in more myopic refractions [44, 45]. In fact, Foulds et al. demonstrated blue light exposure in chicks was able to reverse red light-induced hyperopic changes [46]. Similarly, blue-enriched white light exposure in chicks not only slowed axial elongation but also improved recovery rates from axial elongation due to form-deprivation myopia [47]. Since chick eyes are excellent at transmitting UV light [48], this may be one of the reasons they appear particularly sensitive to blue and violet light treatments. In mouse models, a study by Yang et al. indicated that it was actually red light which induced hyperopic shifts, while blue light appeared to have no role in emmetropization [49]. Conversely, within the same year Strickland et al. published contrary data, where exposure to short-wavelength light in wild type mice induced hyperopia and inhibited myopia development [50]. Short-wavelength light exposure in guinea pig models have shown similar hyperopic shifts [38, 51], as well as demonstrating myopia prevention in lens-induced myopia (LIM) models [52].

This controversy in regard to which wavelength is responsible for myopia prevention across varying species is not new and continues to develop. For example, recently, long-wavelength red light irradiation has emerged as a potential myopia prevention strategy. In 2014, Liu et al. published findings indicating that long-wavelength red light exposure during development in rhesus monkeys could be a potential risk factor for myopia development [53]. However, within the following year, Smith et al. demonstrated that infant rhesus monkeys, when exposed to long-wavelength red light, had a higher prevalence of hyperopia compared to controls [54]. Smith et al.’s findings have been supported by later studies, and it has been suggested that specific ranges of red light may affect myopic progression risk [55]. In tree shrews, Ward et al. reported that red light slowed axial elongation and resulted in hyperopic development. The degree of hyperopia also appears to increase exponentially based on red-light exposure time in young tree shrews [56]. Among primary school children undergoing repeated low-level red-light (LLRL) intervention therapies, He et al. showed a 33.4% relative reduction of myopia incidence [57]. Zhang et al. found a similarly positive influence of repeated LLRL intervention on myopia prevention in children treated for 3 months [58]. A separate study also demonstrated LLRL in children for 6 months resulted in slower axial length, increased choroidal thickness, and reduced refractive shifts compared to both controls and orthokeratology-treated eyes [59]. Since there appeared to be an age-dependent influence on the axial length modification, but no correlation between subfoveal choroidal thickness and age, the authors speculated that the mechanism behind LLRL’s influence on axial growth may not involve direct changes to the choroid [59]. Based on these positive findings, a randomized controlled trial (ClinicalTrials.gov Identifier: NCT04073238) is currently investigating the efficacy of LLRL in regards to myopia prevention.

Although long-wavelength red light has been explored for its myopia prevention potential as previously described, several studies have also indicated that violet light (360–400 nm wavelength) exposure may be the critical component within natural sunlight that is preventing myopia progression [60–62]. This wavelength could be considered of particular interest due to the fact that most modern light-emitting-diode (LED) and indoor lighting omits this wavelength [63], and standard windows are designed to filter out violet light [63, 64]. This could be partially responsible for the epidemiological rise in myopia prevalence and explain, at least in part, why more and more eyes are failing to reach the correct focal length during development. Several studies found that violet light had a consistent effect on myopia development and were able to relate it to several molecular mechanisms and non-visual opsin involvement [60, 65, 66].

To summarize briefly, the majority of evidence for LLRL therapy includes prospective, non-randomized trials in children, with axial length and refractive power as the primary endpoints, and with most follow-ups being less than one year. However, a current ongoing clinical trial may yield more extensive findings regarding the persistence of effects. In contrast, many blue-light therapy studies consist of prospective, non-randomized trials in young adults, with axial elongation being a primary endpoint and limited information on long-term durability. Violet light treatment studies have consisted of both prospective and retrospective studies, mostly in children, with refraction, axial elongation, and choroidal thickness often serving as primary endpoints. Most of these studies have a durability of one year or less, and more long-term studies are needed to verify extended efficacy. For a summary of some of the pertinent animal and human studies of varying spectral-based interventions, see Table 1.

**Table 1:** Spectral interventions characteristics

Study	Species: model	Wavelength/bandwidth	Illuminance	Setting and exposure duration	Stimulation characteristics	Primary endpoints and notable findings
Karouta et al., 2014 [32]	Chicks: FDM	Mix of cool (400–650 nm) and warm (430–700 nm) LED modules; no emission of ultraviolet or infrared spectrum	500 lx, 10,000 lx, 20,000 lx, 30,000 lx, and 40,000 lx	Indoor, 12:12 light:dark cycle; Higher intensities (10,000–40,000 lx) were used for 6 h a day, with normal laboratory lighting (500 lx) for the remaining 6 h	LED bank module: Chicks were exposed to differing intensities of ambient laboratory room lighting	Refraction and AL: Significant correlation between log light intensity and reduced myopia development (*P* < 0.0001) and shorter AL (*P* < 0.0001), with higher light intensities providing more reduction
Seidemann et al., 2002 [44]	Chicks: normal ocular development	Either 615 nm (red, half bandwidth 15 nm) or 430 nm (blue, half bandwidth 15 nm)	Approximately 5 lx intensity adjusted with neutral density grey filters to produce similar brightness despite wavelength differences	Indoor, 12:12 light:dark cycle; 12 h of exposure per day	Large hemispheric dome used to ensure homogenous illumination. Light provided by a 250-W slide projector	Refraction: Chicks reared in normal white light were significantly more myopic (*P* < 0.001) when exposed to 615 nm compared to 430 nm for 2 days. When chicks exposed to 430 nm light for 2 days were shifted to 615 nm light for two days, they became more myopic (*P* < 0.05). When taking chicks from 615 nm light to 430 nm, the trend was reversed and chicks became more hyperopic (*P* < 0.05). When considering all test groups, chicks exposed to 430 nm (blue) light were more hyperopic consistently (*P* < 0.05)
Foulds et al., 2013 [46]	Newborn chicks: normal ocular development	White-emitting LEDs: broad-emission 420–790 nm spectrum, peak at 440 nm and crest at 536 nm; Blue-emitting LEDs: 440–495 nm spectrum, peak at 477 nm; Red-emitting LEDs: 600–680 nm, peak at 641 nm	33.37 cd/m^2^ for red-emitting LEDs; 34.44 cd/m^2^ for blue-emitting LEDs; 117.32 cd/m^2^ for white-emitting LEDs	Indoor, 12:12 light:dark cycle; 12 h of exposure per day	Four banks of LEDs with 30 LEDs per bank fitted into inner side of lid of custom-built rectangular enclosure to ensure uniform distribution of light	Refraction, AL, VCD: Chicks raised in red light were significantly more myopic (*P* < 0.001) than those raised in blue light. Red light induced myopia while blue light induced hyperopia. Average VCD was significantly longer in chicks raised in red light compared to blue light (*P* < 0.001). Trend of increase in mean AL of chicks reared in red light, especially during the first 2 weeks
Yang et al., 2020 [49]	Newborn C57BL/6 J mice: normal ocular development	Quasi-monochromatic red: 585–660 nm; Quasi-monochromatic blue: 410–510 nm; Broad spectrum white light	275 ± 30 lx	Indoor, 12:12 light:dark cycle; 12 h of exposure per day	Prior to eye opening, illumination provided by LED tubes throughout rearing period	Refraction, ERG, and axial dimensions: Mice reared in red light were significantly more hyperopic (*P* < 0.001) than mice raised in white light. Mice reared in blue light had significantly reduced ACD growth compared to white light (*P* < 0.001). AL increased for both red and blue light reared mice compared to white light (*P* < 0.01), and AL growth was less in red light exposed mice compared to blue light exposed mice (*P* < 0.01)
Strickland et al., 2020 [50]	Postnatal day 28 C57BL/6 J mice and Gnat2^−/−^ mice: LIM	Broad-spectrum “white”: 420–680 nm; “green”: 525 ± 40 nm; “violet”: 400 ± 20 nm	Approximately 50 cd/m^2^	Indoor, 12:12 light:dark cycle; 12 h of exposure per day	Custom ventilated light box with LEDs	Refraction and axial parameters: Violet light induced hyperopia and inhibited LIM progression, and the findings were significant compared to white light (*P* < 0.001). Mice housed in green light had no significant differences from white light. No significant differences in AL or VCD between test groups. In Gnat2^−/−^ mice, the effect of violet light was lost
Zou et al., 2018 [51]	Guinea pigs: normal ocular development	Short-wavelength: 430 nm (half-bandwidth 10 nm); Middle wavelength: 530 nm (half-bandwidth 10 nm); White light: normal lighting without particular intervention (broadband, color temperature 5000 K)	Approximately 1770 mW·m^−2^ for blue light, 700 mW·m^−2^ for green light, and 740 mW·m^−2^ for white light	Indoor, 12:12 light:dark cycle; 12 h of exposure per day	Custom cage with walls, ceiling, and floor installed with LED light tubes	Refraction, CC, ACD, LT, AL measured at baseline and after 10 weeks: Mean sphere refraction in short-wavelength group was significantly more hyperopic than white-light group (*P* < 0.01). Refraction of middle-wavelength group was significantly more myopic than white-light group (*P* < 0.01). Short-wavelength group showed reduced AL compared to white-light group (*P* < 0.01), while middle-wavelength group showed longer AL compared to white-light (*P* < 0.01). No other significant differences in other parameters measured
Jiang et al., 2014 [52]	Guinea pigs: LIM	Red light: max wavelength 600 ± 5 nm; Blue light: max wavelength 470 ± 5 nm; White light: fluorescent lamp, color temperature 6500 K	Approximately 300 (human) lux for red light, 50 (human) lux for blue light, and 350 (human) lux for white light	Indoor, 12:12 light:dark cycle; 12 h of exposure per day	Colored LEDs affixed to top of cages; Cage walls, top, and floor all lined with silvered paper to achieve uniform illumination	Refraction, ERG, LT, VCD, AL, choroidal thickness: Red light induced myopic shifts compared to baseline (*P* < 0.01) and increased AL growth compared to white and blue light (*P* < 0.01). Blue light exposure increased choroidal thickness compared to white light (*P* < 0.01). Red light exposed temporarily thinned choroid compared to white light (*P* < 0.01). When considering the LIM model, blue light exposure suppressed myopia (*P* < 0.01) and AL elongation (*P* < 0.01) compared to white light. Red light had no significant effect on LIM model
Liu et al., 2014 [53]	Infant rhesus monkeys: normal ocular development	Quasi-monochromatic blue light: peak 455 nm (half bandwidth 25 nm); Quasi-monochromatic red light: peak 610 nm (half bandwidth 20 nm); White light: color temperature 5000 K	Red light: 0.043 mW/cm^2^; Blue light: 0.14 mW/cm^2^; White light: 0.024 mW/cm^2^	Indoor, 12:12 light:dark cycle; 12 h of exposure per day	Special-built cages with LED tubes installed on walls, ceiling, and floor to obtain uniform illumination	Refraction, axial parameters: Two monkeys in the red light group developed myopia, while the rest remained hyperopic. The mean difference in refraction of the red light group was significantly different from the white and blue groups (both *P* < 0.001), and this change was accompanied by a faster elongation of the VCD. There was no significant difference in the refractions between the blue light and white light groups
Smith et al., 2015 [54]	Infant rhesus monkeys: long-wavelength-pass (red) filters in front of one or both eyes, control reared with neutral density filters or with unrestricted indoor lighting	Standard laboratory fluorescent lighting, color temperature 3500 K	Average of 580 (human) lux	Indoor, 12:12 light:dark cycle; 12 h of exposure per day	Filters worn continuously until 146 ± 7 days of age	Refraction, VCD: Monkeys wearing red filters in front of both eyes demonstrated a significant hyperopic shift compared to neutral density (*P* < 0.001) and normal controls (*P* < 0.001). The hyperopia was associated with shorter VCD. After removal of filters, the monkeys recovered from the induced hyperopia
Gawne et al., 2017 [69]	Infant and juvenile tree shrews: normal ocular development	Red light: peak wavelength 624 ± 10 nm, later replaced with peak wavelength 636 ± 10 nm due to supply shortage; Fluorescent control: color temperature 5000 K	Red light: 527–749 lx; Standard colony illuminance: 100–300 lx	Indoor, 14 h light: 10 h dark cycle; 14 h of exposure per day	Red light produced by narrow-band LED strips placed on top of cage; Fluorescent control produced by compact fluorescent bulbs affixed to top of cage	Refraction, VCD, choroidal thickness: Compared to control, red light exposure resulted in significant hyperopia (*P* < 0.05). In red-treated juveniles, there was also significantly thickened choroid (*P* < 0.05) and reduced VCD (*P* < 0.05) compared to controls
Zhang et al., 2024 [58]	Children aged between 6 and 16 years old: head-mounted low-level red-light emitting device	Red light: 650 nm, single-wavelength weak red-light laser	Average output of 1.44 mW, with power of 0.29 mW entering pupil (radiation category Class 1 light)	Twice daily, 30 min treatment, with at least 4 h between treatment sessions	Head-mounted low-level red-light therapeutic device	Refraction, AL: After 3 months of treatment, average AL was shown to decrease by 0.031, with a non-significant trend for greater reduction in boys. Spherical refraction also decreased by an average of 0.012 ± 0.355 D
Xiong et al., 2021 [59]	Children aged between 6 and 16 years old: LLRL and orthokeratology intervention	LLRL: 650 nm single wavelength	Average output of 2.0 ± 0.5 mW	Twice daily, 3 min treatment sessions, with 4 h between sessions. Ocular parameters measured twice daily, at 8:00 a.m. and 2:00 p.m.	LLRL treatment (no further specifics provided), no specific guidelines for room illumination	Refraction, AL, choroidal thickness: In the LLRL group, mean spherical refraction decreased over time and was significantly different from controls (*P* < 0.001). The mean AL was also shorter than control and orthokeratology groups (*P* < 0.001, both). LLRL group also had increased average choroidal thickness compared to control group (*P* < 0.001)
Torii et al., 2022 [61]	Children between ages 6 to 12: one group wore violet light-emitting eyeglass frames while other group wore pseudo-placebo eyeglass frames	Violet light irradiance of 310 μW/cm^2^	Violet light-emitting frames emitted violet light of 310 μW/cm^2^, while pseudo-placebo frames emitted less than 10 μW/cm^2^ of violet light	3 h of exposure per day, from 11:00 a.m. to 2:00 p.m.	Violet light-emitting frames emitted violet light that was same level of annual average violet light irradiance in Tokyo. Frames also featured violet light sensor to measure outdoor violet light exposure	Visual acuity, tear film break-up time, corneal endothelial cell density, slit-lamp/fundus examination, AL, and refraction: At 6 months, there was a significant difference between AL, choroidal thickness, and refractions for children aged 8–10 years old (*P* < 0.05). In this subgroup, those wearing violet light emitting glasses showed reduced progression of myopia, slowed AL growth, and thicker choroids
Torii et al., 2017 [60]	Chick: FDM and LIM; Children between ages 10 to 18 years old: wearing either violet light blocking eyeglasses, partially blocking contact lenses, or violet light transmitting contact lenses (retrospective study)	For chicks: standard fluorescent light; Violet light: peak 365 nm; Blue light: peak 470 nm	For chicks: average of 1035–1230 lx. Violet light irradiance: 11.191 ± 3.449 W/m^2^; Blue light irradiance: 11.590 ± 3.973 W/m^2^; UV (290–390 nm) irradiance in violet light group: 0.413 ± 0.238 mW/cm^2^; UV irradiance in violet light absent groups: 0	For chicks: 12:12 light:dark cycle; For children: compared baseline measurements to one year later	For chicks: used violet light fluorescent light, blue LED light, and standard fluorescent light bulb	AL, refraction: In chicks, in both LIM and FDM models, myopia eyes exposed to violet light showed significantly less myopia (*P* < 0.001). AL elongation was dependent on amount of violet light transmittance through goggles, with a significant correlation between the amount of violet light transmittance and the myopia suppression (*P* < 0.05). Myopia suppression via violet light was significantly stronger than that provided by blue light exposure (*P* < 0.05). In children, those wearing violet light transmitting contact lenses had significantly reduced AL elongation compared to those wearing violet light blocking eyeglasses (*P* < 0.05)
Jeong et al., 2023 [62]	C57BL/6 J mice: LIM	Violet light: 360–400 nm, peak at 375 nm; White light: 280–780 nm	Background white fluorescent lamp: approximately 50 lx; 40% transmittance allowed 14 μW/cm^2^ intensity, 70% allowed 26 μW/cm^2^ intensity, and 100% allowed 40 μW/cm^2^ intensity	12:12 light: dark cycle; Violet light exposure occurred every day from 18:30 to 20:00	Groups divided into 40% violet light transmittance lens, 70% transmittance lens, or 100% transmittance. Groups were further divided into those receiving 1.5 h of violet light exposure and those who did not	Myopic shift in refractive error, difference in axial length, choroidal thinning: There was a dose dependent relationship between amount of violet light transmission and myopia progression. The groups with 70% and 100% transmission levels had significantly smaller myopia progression in LIM model compared to controls (*P* < 0.01). In these same groups, AL elongation was significantly less than controls (*P* < 0.01). Higher transmittance of violet light provided more prevention of myopia progression
Jiang et al., 2021 [66]	C57BL/6 J mice, *Chx10-Cre;Opn5*^*fl/fl*^ conditional knockout mice: LIM	Violet light (supplementing white background light) LED: 400 μW/cm^2^ (360–400 nm); Blue light LED: 440–480 nm; Green light LED: 500–540 nm; Red light LED: 610–650 nm	Approximately 50 lx white background fluorescent light, color temperature: 5000 K	12:12 light:dark cycle; Violet light added at different times 3 days a week; One group received all day exposure, one group received 3 h of exposure predawn, one group received continuous exposure, one group received 3 h of evening exposure, and one group received 3 h of postdusk violet light exposure	Violet light LED acted as supplement to baseline white background lighting; Received 400 μW/cm^2^ exposure to violet light starting from postnatal day 21; Other wavelengths were compared for efficacy	Refraction, AL, choroidal thickness: Predawn and all day violet light exposure had no significant effect on AL or refraction compared to the while light group. All other test groups showed significant changes in at least one parameter, but 3 h of evening violet light exposure produced the most significant reduction of lens-induced refractive shift and AL elongation compared to white light exposure only (*P* < 0.05). When comparing violet, blue, green, and red wavelengths, violet light showed the most significant reduction of refractive change and AL elongation compared to other wavelengths (*P* < 0.05)
Read et al., 2018 [72]	Young adults between the ages of 20 to 29 years: commercially available light therapy glasses, normal ocular development	Blue-green wavelength: peak 500 nm	506 lx illuminance, 30 min per day	Treatment performed at 7:00 a.m. for 7 days, or whenever the participant woke up if participant did not wake before 7:00 a.m.	Four light emitting diodes directed light towards eye from lower portion of plastic frame, approximately 20 mm from the eye. Also wore wrist-watch light sensor to measure daily light exposure	Macular choroidal thickness: Those who wore light therapy glasses showed significant increase in subfoveal choroidal thickness (*P* < 0.05) after 7 days of daily light exposure
Thakur et al., 2021 [164]	Young adult women aged 20 to 32 years: hyperopic defocus (3 D) induced to one eye	Blue: peak 455 nm (half maximum width = 25 nm); Green: peak 523 nm (half maximum width = 37 nm); Red: peak 620 nm (half maximum width = 35 nm); Broadband white light	Blue light: average irradiance 0.000174 W/nm/m^2^; Green light: average irradiance 0.00021 W/nm/m^2^; Red light: average irradiance 0.00013 W/nm/m^2^	Treatment over 4 different days within a 10-day time frame, 1 h exposure sessions each day	Six light-emitting diode smart bulbs mounted in 3.0 × 2.0 × 3.2 m room. Patients sat within the room and watched a movie on a laptop at 3 m distance, fitted with a cellophane sheet matching experimental light conditions (i.e., red color sheet for red light exposure)	Axial length and choroidal thickness: Red light exposure increased AL from baseline in both defocused (*P* < 0.001) and non-defocused eye (*P* < 0.01), as well as significant decrease in choroidal thickness (*P* < 0.05). Green light exposure also showed significant increase in AL in defocused and non-defocused eyes (both *P* < 0.001), as well as a significant decrease in choroidal thickness (*P* < 0.05). Blue light exposure reduced AL in both eyes (*P* < 0.001 in defocused eye; *P* = 0.11 in non-defocused eye) and showed no significant change in choroidal thickness
Hoseini-Yazdi et al., 2024 [168]	Young adults between the ages of 18 to 35 years: normal ocular development	12 Hz flickering blue light: peak 460 nm; 12 Hz flickering red light: peak 620 nm	Blue light: 22 cd/m^2^; Red light: 139 cd/m^2^	Ambient room lighting 10 lx before and after dark adaptation period; Stimulated for 1 min with blue or red flickering light; Measurements taken over 60-min poststimulation period	Stimulation applied to blind spot while viewing a smart phone through a virtual reality headset. Blind spot was mapped using the virtual reality headset	Choroidal thickness and AL: Compared to controls and red light, blue stimulation increased choroidal thickness (*P* < 0.001). No significant differences in AL
Ellrich et al., 2023 [169]	Young adults between the ages of 30 to 35 years: normal ocular development	Blue light: peak 460 nm; Red light: peak 620 nm	Blue light: 22 cd/m^2^; Red light: 22 cd/m^2^	1 min of blue light exposure over 6 consecutive days. Red light exposure was performed 2 days prior to blue light exposure	Stimulation of ONH while viewing through a virtual reality headset	AL and refraction: There was a significant interaction between blue light ONH stimulation and refractive error for changes in AL (*P* < 0.05), while there were no observed significant changes with red light or no light controls

*ACD* = anterior chamber depth; *AL* = axial length; *CC* = corneal curvature; *D* = diopter; *ERG* = electroretinogram; *FDM* = form deprivation myopia; *LIM* = lens-induced myopia; *LLRL* = low-level red-light therapy; *LT* = lens thickness; *ONH* = optic nerve head; *VCD* = vitreous chamber depth

The theory of longitudinal chromatic aberration (LCA) has emerged as a potential explanation for how these differing wavelengths affect axial growth. This theory relies on the physical principles of light, mostly that a short wavelength of light would focus in front of the retina, therefore encouraging less axial elongation, while a long wavelength would focus behind the retina and stimulate axial elongation. The defocus created in these specific wavelengths could serve as a stimulus for axial growth regulation. For example, if red wavelengths are in better focus than blue wavelengths, the signal would be to slow growth, since the eye was too long to focus blue wavelengths [67]. Conversely, if the blue was in better focus than the red wavelength, then the eye would be stimulated to grow further. Swiatczak et al. confirmed this idea by demonstrating that when a red-blue image was presented with the red wavelength in better focus, there was significant axial length shortening compared to baseline and when the blue wavelength was in better focus (*P* < 0.001) [67]. Participants were shown the same movie in three separate scenarios. In one scenario, the movie was presented with the green and red channels low pass filtered so that the blue channel was sharp, while in a second scenario the movie was presented with the green and blue channels filtered so that the red channel would be sharpest. For the control scenario, the unfiltered movie was presented. When measuring changes in axial length in 15-min intervals throughout the movie viewing session, ocular growth elongation was significantly increased when viewing the movie with the blue channel in focus (*P* < 0.01) [67]. Gawne et al. demonstrated that isolating and blurring the blue channel of a video display resulted in a significant hyperopic shift due to reduced axial growth compared to controls (*P* < 0.0001) [68]. It is important to note that for the study of Swiatczak and Gawne, spatial frequency patterns and low pass filters were used to create the defocus, rather than true optical defocus. This hypothesis of LCA is sound in theory, but when one starts to see the variation in results between species as to whether long- or short-wavelengths of light inhibit myopia, it becomes apparent that the mechanisms at play are complex and likely influenced by the entire visual spectrum. It is possible that certain species might show spectral sensitivities to particular wavelengths. For example, there may be several cone types contributing to the process of emmetropization [44]. Rucker et al. also suggested that the range over which LCA might influence emmetropization is limited and might only be at play when refraction is hovering around emmetropia [45]. For this reason, it may be that there are other defocus cues besides just LCA signals that influence the eye’s compensation to a large range of myopic and hyperopic defocus. In a separate tree shrew study to the previously mentioned, Gawne et al. showed in tree shrews that a flickering short-wavelength blue light resulted in myopia progression but a steady blue light allowed normal emmetropization, and in the process pointed out how the experimental environment was very different from a natural environment, in which the LCA cues would change over time [69]. This highlights a notable limitation to many of these studies: they create an open-loop feedback system. The wavelength stimulus stays constant regardless of how the eye responds. Realistically, there is more likely a closed loop feedback system, where the eye’s emmetropization mechanisms respond to the stimulus and balance changes over time. In addition, some of these studies are confounded by the fact that the effects of defocus and color information are intermixed, so it is challenging to attribute ocular changes to only the wavelength properties of the light tested or the varying color vision of the animals being tested. There may also be varying distributions in retinal photoreceptors and species-specific sensitivities to particular spectrums of light which could explain the varying results, but more research is needed.

These differences emphasize the critical need when considering light therapies as potential myopia prevention strategies to also consider the light intensity, pattern timing, and potential influence on the circadian rhythm. For example, it has also been proposed that increased light intensity alone can reduce myopia. In several studies of school children, children exposed to higher intensities of light showed less myopia development [70, 71]. When wearing light therapy glasses emitting around 500 lx light for 30 min a day for 7 days, there were significant increases in choroidal thickness reported [72]. These findings have also been supported by studies in tree shrews [73], chicks [74], and rhesus monkeys [34] with form deprivation myopia demonstrating reduced axial length and myopia when exposed to increased environmental lighting.

More in-depth, comprehensive molecular analyses of what results from exposure to certain wavelengths of light are warranted to better understand myopia progression in relation to visual cues.

The non-visual opsins have been identified as having peak absorption ranges in the blue to violet light range [75, 76]. As such, the demonstrated relationship between violet light and myopia led to the question of whether the non-visual opsins might be at least partially responsible for some of the ocular growth cues in response to light exposure in this spectrum. Preliminary research, which will be discussed below, appears to support the hypothesis that the non-visual opsins are stimulated by violet light exposure to embrace myopia-preventing measures, and thus reveal promising and exciting new opportunities for intervention strategies.

### OPN5 (Neuropsin)

Mouse and human OPN5 show a maximum absorption (λ_max_) at 380 nm, squarely within the violet light range [77]. It is worth noting that this peak sensitivity lies within the UVA radiation spectrum as defined by the World Health Organization (WHO) [78]. Due to this wavelength being on the shorter spectrum, it is often blocked by modern filters on windows and lenses, as well as being limited in artificial lighting sources. A recent study by Kondo et al. demonstrated there is a notable lack of violet light in indoor environments [63], indicating that while OPN3 and OPN4 may be activated indoors, there is a notable deficiency in OPN5 activation. OPN5 has been identified not only within the eye but also in the brain, testes, spinal cord, and, perhaps surprisingly, in the outer ears of mice [77, 79]. When exposed to violet light, OPN5 activates heterotrimeric G protein Gi, thereby inhibiting adenylyl cyclase and reducing cAMP levels [77, 80]. Recently, it has also been demonstrated in humans that OPN5 activates Gq-type G protein [81]. Additionally, OPN5 has been implicated in circadian clock entrainment [82, 83] as well as light-dependent vascular development within the eye [84]. In regards to the vascular development, Nguyen et al. demonstrated a lack of OPN5 results in erratic regression of hyaloid vasculature, which is crucial for establishing a clear optical axis for visual function [84]. These researchers suggested this may be due to violet light increasing the activity of the dopamine transporter (also called SLC6A3, a dopamine reuptake transporter) via OPN5 and the vesicular GABA/glycine transporter, which in turn suppresses vitreous dopamine. When OPN5 function is disrupted, vitreous dopamine levels elevate, which suppresses the activity of vascular endothelial growth factor receptor 2 (VEGFR2) and promotes premature hyaloid vessel regression, but retinal dopamine levels actually decreased [84]. Moreover, dopamine levels were shown to be modulated by stimulation with 380 nm violet light [84]. It is worth noting this study primarily focused on P1 to P8 mouse pups before their eyes opened. Whilst there are no studies in humans confirming these findings, the observation that premature infants treated with dopamine have a higher risk of retinopathy of prematurity suggests there may be a similar vascular development relationship in humans [85, 86]. As OPN5 is highly conserved, it is possible that the OPN5-based dopamine pathway’s influence on vascular development persists in human infants, but this is still speculation at this point. Although there is no definite evidence of OPN5 expression within dopaminergic amacrine cells in humans, there is data from chickens of OPN5-positive cells being within close enough proximity to dopaminergic amacrine cells to have dendritic interactions [80]. In chicks, it has also been found that there are wavelength-dependent differences in retinal dopamine release, with blue and violet light promoting increased levels [87]. Guinea pigs have shown similar increases in retinal melanopsin mRNA and protein expression when exposed to 480 nm blue light vs. white light, which influences dopamine signaling, in addition to being less myopic [88]. In rabbit models, blue light stimulation resulted in increased dopamine levels in the aqueous humor and vitreous body [89].

Dopamine has been implicated in myopia development pathways for over 30 years [90], but in recent years it has been receiving more attention as researchers try to uncover strategies to mitigate the global increase in myopia [91]. Retinal dopamine is generally regarded as a cue to the eye to cease myopic axial elongation, and both form deprivation myopia and LIM models have shown reduced retinal dopamine levels [92]. In chick models, increased dopamine levels were significantly correlated to increased choroidal thickness (*P* < 0.01) [93]. Some studies attribute retinal dopamine release solely to OPN2 [94], but others demonstrate that OPN5 [84, 95] and OPN4 [96] are also related to dopamine regulation. Questions about OPN5’s potential role in myopia development naturally began to arise. In addition, given that OPN5 has a maximum absorption within the violet light range [77], and previous studies demonstrated the preventative potential of violet light exposure in regard to myopia progression [60], a relationship between OPN5 and myopia became even more plausible.

This role was tested and confirmed by Jiang et al., where stimulation of *Opn5* retinal ganglion cells (RGCs) with short-wavelength violet light prevented experimental myopia in LIM mice [66]. When supplementing white fluorescent light with blue (440–480 nm), green (500–540 nm), red (610–650 nm), or violet light (360–400 nm) at the same irradiance level, violet light yielded the strongest myopia suppression effect in LIM mice [66].

*Opn5* expression in the retina appears to be limited to a particular subset of RGCs [66, 82, 97]. When Jiang et al. selectively deleted *Opn5* specifically from the retina using *Chx10-Cre; Opn5*^*fl/fl*^ mice, this resulted in the loss of the violet-light dependent suppression of myopia in the LIM model [66, 98].

Upon further exploration of the potential underlying mechanisms by which OPN5 modulates myopia, it was discovered that the influence of violet light on choroidal thickness also appears to be dependent on OPN5 [66]. In both humans [99] and animal models [100], myopic eyes demonstrate notably thinner choroids. It is believed these changes in choroidal thickness may be a major influence on axial elongation and refraction. The choroid serves as the major vascular supply of the eye, and changes in thickness might reflect changes in choroidal blood flow. Seeing as hypoxia is related to myopia, changes in choroidal thickness may result in alteration of oxygen and nutrient supply, which in turn may trigger scleral ischemia and initiate structural alterations that ultimately lead to myopia through axial elongation [101]. This idea is further supported by the fact that choroidal thinning appears to precede axial elongation [101]. Violet light has been shown to have a protective effect on choroidal thinning in a myopic setting. For example, Torii et al. and Mori et al. demonstrated children wearing violet light-emitting glasses had reduced axial elongation and maintained choroidal thickness [61, 102]. Ogawa et al. also demonstrated that children who participated in intensive outdoor activity for one week showed increased choroidal thickness, possibly due to increased time spent in natural sunlight containing violet light [103]. Ex vivo studies have indicated that violet light causes an expansion in the choriocapillaris, which may be fundamental to maintaining both choroidal thickness and sufficient oxygen supply to surrounding tissues, which may prevent myopic structural changes [104].

As previously highlighted, Jiang et al. demonstrated that in *Opn5-*conditional mutant mice (*Chx10-Cre;Opn5*^*fl/fl*^) subjected to the LIM model, supplementation of violet light was no longer able to elicit a protective effect against myopia progression [66]. These mice also demonstrated notable choroidal thinning even with violet light exposure, while wild type mice receiving violet light supplementation showed no notable choroidal thinning [66]. This gives further evidence that the changes in choroidal thickness seen with violet light exposure are dependent upon an OPN5 pathway. Given its role in other vascular development within the eye [84], there was little doubt that OPN5 could also influence the highly vascular choroid.

Choroidal thickness also appears to be influenced by circadian rhythm [105], which in turn is heavily influenced by lighting changes, including violet light [106, 107]. The circadian clock is known to play a key part in retinal function, such as through regulating light sensitivity during day and night [108] and maintaining photoreceptor outer-segment disk membranes [109]. Circadian rhythm has also been previously indicated to influence the refractive development of the eye, and retina-specific disruption of the clock gene *Bmal1* in mice leads to myopic refraction, longer axial length, and vitreous chamber elongation [110]. Study findings like those of Osol et al. and Li et al. in chicks exposed to varying cycle amounts of light and dark have indicated that at least 4 h of light and 4 h of darkness are needed for emmetropization to occur [111, 112], indicating the need of a light cycle for normal eye growth. OPN5 is critical for the establishment of circadian rhythm within the mouse retina [82], and retinas lacking OPN5 have demonstrated the inability to photoentrain [83]. However, while these OPN5-null mice have impaired photoentrainment, it does not appear to be a complete loss of function [83], making it difficult to draw conclusion about the underlying mechanisms. A study by Buhr et al. also indicated OPN5 is necessary for murine retinal and corneal circadian clock photoentrainment ex vivo [113]. The mechanism by which violet light suppresses myopia via OPN5 may involve the regulation of the retinal circadian clock. For this reason, studies have suggested that the timing of violet light exposure to prevent myopia may be important. For example, Jiang et al. propose that supplementing with violet light at the beginning of the day cycle may have the greatest influence on myopia suppression [66].

Violet light exposure also results in the upregulation of early growth response-1 (EGR-1) expression, and the response appears to be dose dependent [60]. EGR-1 is a key player in both ocular growth and refraction [114–116]. Jeong et al. demonstrated that violet light created the most robust elevation in EGR-1 expression when compared to other wavelengths [65], which is consistent with findings that violet light is also the most effective in myopia suppression [66]. In turn, this violet light-induced EGR-1 expression in the retina has been shown to be mediated by OPN5 [65]. Specifically, while wild-type mice show a significant increase in *Egr-1* mRNA expression 7 h after violet light exposure (*P* < 0.001), *Opn5* knockout mice showed little increase in mRNA expression of *Egr-1* in the retina following violet light exposure [65]. In regards to EGR-1 protein expression, while 661W cells demonstrated a significant increase 1 h after violet light exposure (*P* < 0.05), there was a much more muted response in an *Opn5* knockout of 661W cells 1 h after violet light exposure compared to controls (*P* < 0.05) [65]. In retina-specific Opn5 knockout mice exposed to violet light for 3 h, there was a significant reduction in both EGR-1 mRNA expression and protein expression in the retina 7 h after exposure compared to controls (*P* < 0.05) [65]. Similar to the limitations of studies attempting to draw conclusions about LCA, this study environment did not replicate a truly natural setting, and so it is difficult to confidently state whether broad spectrum daylight is able to elicit a similar alteration in EGR-1 mRNA and protein expression levels. However, it provides some indication of OPN5 being a potential underlying mechanism in ocular growth alterations due to violet light exposure. In chick fibroblasts, Kato et al. demonstrated OPN5 was responsible for upregulation of *Egr-1* expression following violet light (375 nm) exposure [117]. This violet light-OPN5-ERG-1 pathway remains a promising area of future research, with some researchers already hypothesizing that retinal dopamine may again be a critical component in this circuit [65, 84]. Nevertheless, it is important to keep in mind it has been well documented that EGR-1 is regulated by a wide range of stimuli, so attributing its regulation solely to Opn5 stimulation is unlikely and it may also only be one of many possible downstream mediators. More research is needed to fully elucidate the mechanisms behind OPN5’s involvement in myopia, but great strides have already been made towards understanding its role.

### OPN4 (Melanopsin)

As mentioned previously, OPN4 has received a fair amount of attention from researchers compared to OPN3 and OPN5, although its contribution to myopia development is still relatively unexplored. It was first discovered in the hypothalamus, retina, and iris of Xenopus laevis in 1998 [118]. Two years later, it was identified in the human inner retina [119]. Its role in the regulation of circadian rhythm is well established, although there are variations shown in different vertebrates [76]. This melanopsin-associated photoreceptive system exists separately from the rod-cone system, relying on intrinsically photosensitive retinal ganglion cells (ipRGCs) [76, 120], but it is well established that rods and cones communicate with ipRGCs and their connections allow for simultaneous contributions to various accessory visual functions [76, 120]. OPN4’s peak sensitivity falls around 480 nm [120, 121], however there appears to be some variation based on measurement method and species [122].

OPN4’s role in myopia development is less understood than its contribution to circadian rhythms and pupillary light reflexivity, but it does appear to have influence. In constant darkness, *Opn4*^*−/−*^ mice show no evidence of a disrupted circadian rhythm or difficulties photoentraining, but when exposed to short pulses of monochromatic light they demonstrate a reduced phase reset ability [123]. These findings indicate that while photoreceptor-mediated communication can still occur in the absence of OPN4, OPN4 is a component of circadian photoentrainment in mammals. For example, Chakraborty et al. found that *Opn4*^*−/−*^ mice that lack melanopsin photopigments and thus have no responses from ipRGCs, but still receive other photoreceptor-mediated communication, demonstrate altered refractive development and a greater myopic shift in response to form deprivation myopia when compared to *Opn4*^+*/*+^ wild type mice [96]. *Opn4*^*DTA/DTA*^ mice, which lack ipRGCs responses due to ipRGC death, also showed greater myopic shifts in a form deprivation model [96]. However, it is worth noting that a study by Liu et al. in 2022 found opposing results. In their study, *Opn4*^*Cre/Cre*^ mice demonstrated reduced axial length and hyperopic refractions compared to wild type controls [124]. In addition, when ablating ipRGCs using melanopsin-saporin (MEL-SAP), an immunotoxin, the myopic shifts induced by form deprivation were notably smaller than those in control mice [124], rather than larger as seen with the *Opn4*^*DTA/DTA*^ mice of the Chakraborty et al. study [96]. The two studies may have come to different findings due to differences in methodology, such as different strategies for ablating ipRGCs, different time periods for form deprivation, and variations in how control groups were assigned. As it stands, more studies are needed to resolve these conflicting results.

It was hypothesized that ipRGCs may influence myopia progression through a dopamine-dependent mechanism. A number of studies have supported the hypothesis that light-stimulated dopamine regulates myopia development [31, 34, 74, 125]. As mentioned in previous chapters, there is a growing body of evidence connecting myopia development to retinal dopamine levels [126].

Supplementation with the dopamine precursor levodopa (L-DOPA) has proven to decrease myopia susceptibility in form-deprivation models across several different species [127, 128]. When *Opn4*^*−/−*^ mice with form deprivation are treated with L-DOPA, the induced myopia is reduced by half compared to untreated mice [96]. These results suggest that OPN4 plays a role in myopia development via ipRGCs and dopamine regulation, and the rate and severity of normal refractive development is influenced by disrupted retinal melanopsin signaling. However, it is worth noting that several studies have found that increasing dopamine signaling or providing exogenous L-DOPA have no influence on myopia development when there is normal, unimpeded visual input [128–130]. This suggests that dopamine’s role may be most influential when the eye is exposed to challenging, unideal visual conditions during development. Therefore, it may play a role in specific protective processes initiated when normal emmetropization is disrupted. If baseline dopamine levels were pertinent for appropriate ocular growth and development, one would expect that eyes with decreased levels of retinal dopamine would be at greater risk to develop myopia, but study findings have been unable to fully confirm this. When creating an experimental model with pharmacologically reduced dopamine levels in chickens, form deprivation myopia was actually reduced and axial length growth reduced [131, 132]. In contrast, studies by Wu et al. and Bergen et al. utilizing wild-type mice with pharmacologically depleted dopamine and mice with retina-specific dopamine knockouts found that reduced retinal dopamine did result in relative myopic refractive errors, but this finding was mostly due to a steepened cornea, and the axial length was actually shortened [133, 134]. In chickens, retinal levels of 3,4-dihydroxyphenylacetic acid (DOPAC), the primary retinal metabolite of dopamine, decreases locally in response to form deprivation myopia [90]. However, in wild-type mouse models of form deprivation myopia, retinal dopamine levels remain unaltered [135, 136]. These differences in findings may also be attributable to species-specific differences in dopamine circuitry. For example, birds and reptiles lack the dopamine transporter and instead rely upon the noradrenaline transporter gene [137], which can result in unique dopamine reuptake activity. Recently, some studies have emphasized the importance of considering the retinal DOPAC/dopamine ratio when considering myopia development [135, 138], rather than simply dopamine levels, as it can provide slightly different inferences than just retinal dopamine levels alone, which indicate direct output. The DOPAC/dopamine ratio represents the amount of dopamine turnover, rather than dopamine release. In C57BL/6 mice, Shu et al. reported retinal dopamine levels were unaffected in form deprivation-induced myopic mice, but the retinal DOPAC/dopamine ratio was decreased [139], indicating an important distinction in these measures. Similarly, Park et al. found the DOPAC/dopamine ratio was more closely correlated with larger myopic shifts than dopamine [140]. The regulation of retinal dopamine does not seem to be the only component contributing to visually-driven ocular growth and development, and there may be variances depending on the species’ dopamine circuitry. This relationship appears to be much more complex and requires further study in order to fully elicit its role.

### OPN3 (Encephalopsin)

OPN3 was first identified in the adult mouse brain over 25 years ago [141], and although it is the most abundantly expressed non-visual opsin in mammals, the breadth of its functional significance is still not fully elucidated; it has only very recently emerged as a potential contributor to myopia development. Unlike OPN4 and OPN5, OPN3 has received only a smattering of research attention even though it is the most abundantly expressed. *Opn3* expression has been detected in a wide range of tissues, including the brain, heart, eye, tooth, skin, lung, adipose tissue, and the immune system [142], as well as within the mouse retina [143]. OPN3 shows a maximum absorption sensitivity of around 465 nm [144], within the blue light range. OPN3 has also been shown to have light-independent functions, which might explain its widespread distribution, including in tissues that are not readily exposed to light [142, 145]. These light-independent functions are not unique to OPN3, and have been described for a number of opsins, including rhodopsin [146]. OPN3 has been implicated in a number of these light-independent systemic processes, including regulating skin melanogenesis [145], metabolism [147], muscle relaxation [148], and immunological processes, such as T-cell signaling in asthma [149]. OPN3 has also been identified as a potential mechanism for cutaneous wound healing, which demonstrates a new potential of light-based therapies for healing skin lesions [150]. In mice, Nayak et al. showed that adipocytes utilize an OPN3-dependent light sensitivity to regulate body temperature and energy metabolism [151].

As previously mentioned, OPN3’s role in myopia is still mostly unresearched. Within the eye, *Opn3* is expressed in both the retina and the choroid, although only in a restricted number of cells in the choriocapillaris for the latter [152]. Myopia-related genes, including *Ctgf, Cx43,* and *Egr-1*, show expression changes dependent on OPN3 [152]. For example, in both LIM models of the tree shrew [153] and mouse [152], retinal *Ctgf* expression levels were significantly downregulated (*P* < 0.05 for both studies). Similarly, retinal *Egr-1* was downregulated in myopic guinea pigs [115], and *Egr-1* knockout mice have been used as a genetic model of induced myopia [114]. As mentioned previously, EGR-1 expression is light-dependent and mediated at least in part by OPN5, and OPN3’s downregulation of *Egr-1* mirrors that seen in other myopia models and indicates a possible downstream mediator by which OPN3 influences refractive development.

Interestingly, a notable number of RGCs expressing *Opn3* overlap with OPN4 RGCs. Specifically, the OPN4 RGC subtypes believed to be involved in refractive development and myopia, M2 and M4, also show *Opn3* expression [152]. This was perhaps the first hint that OPN3, like OPN4 and OPN5, may also play a role in normal refractive development. This same study by Linne et al. was unable to detect any overlap between *Opn3* and *Opn5* expression with RGCs [152]. While this suggests that OPN3 and OPN4 might function in relation to each other, OPN5 RGCs are likely a separate population, despite OPN5 and OPN3 both influencing EGR-1. Deletion of retinal *Opn3* does not influence the expression of OPN4, and in fact their respective dysfunction leads to separate myopic phenotypes [152].

When comparing OPN3 retinal and germline mutations in a mouse model, it is only the OPN3 germline mutation that results in a refractive myopia phenotype, exhibiting a thinner lens and shorter axial length due to reduced anterior chamber depth, in contrast to typical axial myopias [152]. Changes in lens thickness to account for axial length alterations during refractive development is a normal component of emmetropization, and failure to do so has been linked to myopia development [154]. However, lens thickness and power are not always connected, and in fact the relationship is often considered paradoxical [155]. It has been demonstrated that a thinner, but not displaced, crystalline lens may be an indicator of myopia [156]. Meanwhile, *Opn4* null mice show slowed axial growth [124] and no change in lens thickness, although the lens is often displaced [96]. Despite the fact that there appears to be notable overlap in *Opn3* and *Opn4* expression within ipRGCs, deletion of *Opn3* from the retina fails to produce any myopic phenotype [152]. Linne et al. were only able to obtain a myopic phenotype with a germline mutation of OPN3 [152]. This somewhat surprisingly indicates that the OPN3 response resulting in refractive changes is not likely to be located within retinal neurons but is originating from a non-retinal tissue. As such, there are still many questions that remain and more research into the separate roles of the non-visual opsins in relation to myopia is needed. Table 2 provides a general summary of the findings of the non-visual opsins in relation to refractive error, ocular parameter changes, genes of interest potentially involved in signaling pathways, and possible relations to dopamine regulation.

**Table 2:** Summary of non-visual opsins in relation to refractive changes, ocular parameter changes, genes of interest, and potential relationship to dopamine regulation

Non-visual Opsin	Refractive error	Ocular parameters	Genes of interest potentially involved in non-visual opsin signaling	Dopamine relation
OPN5	*Opn5* deletion from retina results in myopic shift in LIM model [66]	OPN-5 conditional mutant mice (*Chx10-Cre;Opn5*^*fl/fl*^) subjected to LIM demonstrate thinner choroids and longer axial length [66]	*Egr-1* [65] *Bmal1* [110]	Disruption results in elevation of vitreous dopamine levels [84] Retinal dopamine modulation influenced by blue and violet light exposure [84, 87–89]
OPN4	*Opn4*^*DTA/DTA*^ mice and *Opn4*^*−/−*^ mice show large myopic shifts in FDM model [96] *Opn4*^*Cre/Cre*^ mice with FDM demonstrated hyperopic refractions [124]	*Opn4*^*Cre/Cre*^ mice with FDM demonstrated slowed axial length [124], *Opn4*^*−/−*^ mice with FDM demonstrated lens displacement but no change in lens thickness [96]	*Bmal1*[110]	FDM-induced *Opn4*^*−/−*^ mice have reduced retinal dopamine and DOPAC [96]
OPN3	Myopic shifts, but only observed with germline mutation of OPN3; Retinal OPN3 deletion yields no refractive changes in LIM mouse model [152]	OPN3 germline mutation leads to thinner lens, shorter axial length due to reduced anterior chamber depth [152]	*Egr-1, Ctgf, Cx43*[152]	Not currently known

*DOPAC* = 3,4-dihydroxyphenylacetic acid; *FDM* = form deprivation myopia; *LIM* = lens-induced myopia

### Treatments based on non-visual opsin findings

The increased incidence of myopia and the severe ocular impairments related to it drive the demand for strategies to both treat and prevent severe myopia. It is especially of interest to find intervention strategies that are suitable for children, as this is when the onset of myopia occurs and often progresses the fastest. Quick and timely intervention strategies with high effectiveness are unfortunately scarce. Currently, strategies to prevent myopia include topical drugs such as atropine [157], orthokeratology lenses [158], low-intensity light therapy [59], multifocal eyeglass lenses and contact lenses [159], and defocusing eyeglass lenses and contact lenses [160]. These treatments are often unable to completely suppress myopia and compliance can occasionally pose an issue. A recent systematic review of different optical interventions for myopia control indicated that the efficacy of treatments reduced over time, with the highest impact being within the first year of treatment [161]. As more mechanisms of myopia are discovered, including those related to OPN3, OPN4, and OPN5, new therapeutic options are being developed that may yield better adherence rates and improved effectiveness. It may be that a combination of currently established treatment methods and newer treatments based on non-visual opsins may provide the best defense against myopia progression, but more research on the combination of therapies is required before this can be determined.

Non-visual opsins may help drive some of the currently emerging promising therapeutic strategies to mitigate myopia progression. For example, the role of EGR-1 in myopia development inspired Mori et al. to investigate dietary factors that could induce EGR-1 activity [162]. EGR-1 has been implicated in both OPN5-mediated light-dependent myopia [60, 65] as well as OPN3-dependent refractive myopia [152], and so its modification may yield promise as a myopia control pathway. Crocetin, a secondary metabolite of gardenia fruit [163], has been identified as a dietary factor that can activate EGR-1 in a dose-dependent manner [162]. When crocetin was fed to a murine model of LIM, both myopic refraction and axial elongation changes were mitigated [162]. Crocetin administration also prevented the choroidal thinning characteristics of LIM murine models [162], but whether this is directly related to EGR-1, and in turn any relation to non-visual opsins, remains to be determined.

One promising therapeutic strategy that has emerged from the study of non-visual opsins is the mitigation of myopia development through exposure to violet light (360–400 nm). While LLRL may emerge as a promising preventive myopia strategy based on the findings of currently ongoing clinical trials and future studies, it has yet to be shown to have a direct relation to the non-visual opsins. However, violet light’s protective effect on myopia development appears to hinge upon OPN5 [66]. Since OPN5 in the human retina has an almost identical absorption spectrum as mouse OPN5 [66], one would expect these results to be generalizable to a clinical setting. In fact, in humans, short-term exposure to red and green light led to axial elongation, while long-wavelength blue light exposure halted axial elongation [164]. Torii et al.’s retrospective study comparing violet light-blocking and violet light-transmissive contact lenses found that violet light-transmissive contact lenses reduced myopia development [60]. In adults, phakic intraocular lenses that permit violet light transmission have also been shown to reduce axial length elongation and myopia progression when compared to phakic intraocular lenses that block violet light [165]. Brainard et al. previously demonstrated that children under the age of 10 have a higher transmission rate of ultraviolet radiation through the lens [166], which indicates that therapy may be even more effective in children. In one clinical trial conducted by Torii et al., wearing violet light-emitting eyeglass frames for 3 h per day resulted in significant changes in choroidal thickness, axial elongation, and cycloplegic refraction in children aged 8 to 10 years old when compared with a control group (*P* < 0.05 for all measures) [61]. The reduction in axial elongation was modest, with around 20% reduction over 2 years [102], indicating that this treatment may best benefit from combination with other established treatment strategies for maximum myopia control. These glasses were developed by The Tsubota Laboratory, Inc. (Tokyo, Japan) in conjunction with NEWOPTO Corporation (Kanagawa, Japan), and emit a violet light irradiance of 310 µW/cm^2^ (Patent No. 6175210) [61]. In addition, there appears to be no significant safety concerns to this treatment. Based on murine studies, violet light exposure at dawn may be the most effective in preventing myopia [66]. These studies have already yielded promising results with acceptable safety profiles, indicating an exciting new potential for future treatment options. Compared to red-light therapy, however, violet light research is limited, and as such there are no comprehensive systematic reviews or meta-analysis to fully confirm effectiveness. Furthermore, both the studies by Torii et al. and Mori et al. reported some difficulty with compliance and follow-up [61, 102], indicating that there may be a need for more robust experimental findings to assure participants of the safety and efficacy of the treatment for future clinical trial attempts.

Blue-light stimulation therapy is another emerging myopia prevention strategy. In young adults, short-term exposure to a full-field blue light reduced the effects of a 3-diopter hyperopic defocus and slowed axial elongation [164]. As ipRGC axons converge at the optic disc, the physiological blind spot created by the optic disc may serve as a more useful target for light-based therapies. By targeting blue-light stimulation at the blind spot, one can ideally stimulate the majority of ipRGCs while limiting the potential effects involving rods and cones [167]. When using blue light to activate OPN4 expressed within ipRGCs within the optic nerve head of rabbits, it was possible to modulate dopamine levels in the posterior segment [89]. Since the light is administered at the physiological blind spot, this treatment is barely perceptible to the patient [167]. Blue-light stimulation at the blind spot resulted in increased choroidal thickness [168] and reduced axial elongation [169] in young adults. Finally, while L-DOPA treatment or other modulations of retinal dopamine levels could potentially be a promising therapeutic option for preventing myopia development, there is not yet conclusive evidence to fully support it. In both form deprivation and LIM chick models, topical L-DOPA administration slows ocular growth and mitigates myopia development with a similar dose-dependency to that seen in topical atropine administration [170, 171]. Chakraborty et al. successfully demonstrated that L-DOPA supplementation was able to prevent myopia induced by form deprivation in *Opn4*^*−/−*^ mice, but it could not prevent naturally occurring myopia in *Opn4*^*−/−*^ mice [96]. This might be due to the fact that Chakraborty et al. only tested a single dose at one concentration (1 mg/mL), and thus future studies comparing the efficacy of higher doses is warranted [96]. Although prior preclinical studies have shown varying results, the potential therapeutic value of L-DOPA should not be dismissed. The results of current ongoing clinical trials may demonstrate more concrete findings to help understand L-DOPA’s potential therapeutic role in ocular development.

## Conclusions

The non-visual opsins have been slowly and steadily gathering more attention in recent decades, and their role in myopia development is still being defined. While much more remains to be explored, what has come to light has yielded promising research acumen. The non-visual opsins offer exciting new explanations for the epidemiological evidence of the protective effects of outdoor sunlight against myopia development and provide insight into potential strategies to mitigate myopia progression. The relationship between OPN5 and myopia includes connections being made between OPN5’s sensitivity to violet light triggering subsequent myopia mitigating phenotypes, possibly in part due to changes in EGR-1 modulation, but it is unlikely this is the only way OPN5 influences myopia development. In addition, OPN3 has been shown to influence EGR-1 regulation, but its mechanism appears separate from that of OPN5. It remains to be seen whether there is any interplay between these two non-visual opsins, or whether they have distinct but redundant capabilities that necessitate further explanation. OPN3 perhaps remains the most mysterious of the non-visual opsins, since despite its widespread distribution very little is known about its role. Meanwhile, OPN4’s role has been explored more extensively, and there is a better understanding of its contributions to circadian rhythm. Its role in myopia development, however, is still being determined. It appears that the regulation of retinal dopamine may be involved, but until more consistent findings emerge it is difficult to draw any solid conclusions.

Based on some of the non-visual opsin research findings, potential future applications, both in and out of clinic, may be on the horizon. New lighting regulations and window transmission rates may result from studies of the potential beneficial effects of violet light. Similarly, dependent on future studies to confirm efficacy and safety, red light emitting or blue light emitting devices, or perhaps even both, may be utilized within clinical practice on a regular basis to prevent myopia development. There may even be some indication for at-home wearable devices, such as wavelength-specific light-emitting eyeglass frames. In combination with future studies elucidating the mechanisms and mediators involved with non-visual opsins, more long-term prospective and clinical trials for light-based therapies’ role in myopia prevention are called for.

In summary, the non-visual opsins’ ability to regulate myopia development poses them as an exciting research topic in these modern times, as the global prevalence of myopia continues to rise dramatically. By discovering their underlying mechanisms, non-visual opsins may eventually help facilitate treatments to prevent high myopia and reduce the burden of visual impairment.

## Data Availability

Not applicable.
